# Aerobic Exercise Improves Depressive-like Behavior in CUMS-Induced Rats via the SIRT3/ROS/NLRP3 Signaling Pathway

**DOI:** 10.3390/life13081711

**Published:** 2023-08-09

**Authors:** Lijun Wang, Yuanyuan Liu, Tuo Xu

**Affiliations:** School of Physical Education, Shaanxi Normal University, Xi’an 710119, China

**Keywords:** aerobic exercise, depression, CUMS, SIRT3, ROS

## Abstract

Objective: This study aimed to investigate the effect of exercise on depressive-like behavior induced by chronic unpredictable mild stress (CUMS) in rats and to explore the role of the SIRT3/ROS/NLRP3 signaling pathway in this process. Methods: Twenty-nine male 8-week-old Sprague Dawley rats were divided into a control group (CON) (nine rats) and a model group (twenty rats). Thirteen chronic stress stimuli were randomly applied once or twice per day for 35 days to induce depression in the model group rats. After the model was established, the model group rats were randomly divided into the CUMS group (CUMS) and the aerobic exercise + CUMS group (EX + CUMS). The EX + CUMS group received 8 weeks of aerobic exercise intervention for 6 days per week. Behavioral assessments were performed using the sucrose preference test and forced swimming test. The expression of SIRT3, NLRP3, IL-1β, and IL-18 in the hippocampus was detected using RT-PCR. The ROS level in the hippocampus was detected using immunofluorescence. The protein levels of SIRT3 and NLRP3 in the hippocampus were detected using western blotting. The protein levels of IL-1β and IL-18 in the hippocampus were measured using ELISA. Results: After 5 weeks of chronic stress stimuli, the hippocampal function of rats in the CUMS model group was impaired, and their sucrose preference was reduced, while their forced swimming time was prolonged. The expression of SIRT3 decreased, ROS increased, and the expression of NLRP3 and the levels of IL-1β and IL-18 increased. Aerobic exercise increased the sucrose preference of rats, shortened their immobility time, increased the expression of SIRT3, and reduced the levels of ROS, NLRP3, IL-1β, and IL-18. Conclusion: Exercise can improve the depressive behavior of CUMS model rats, and its mechanism may be related to the upregulation of SIRT3 in the hippocampus, which plays an anti-inflammatory role.

## 1. Introduction

Depression has long been recognized worldwide as an important cause of mental and physical disability [1,2], and the WHO indicates that approximately 280 million people in the world suffer from depression [3,4]. Symptoms of depression include loss of interest and pleasure, sleep disturbances, fatigue or low energy, and suicide attempts [5]. Adverse outcomes associated with depression include recurrence of depression; episodes of other psychiatric disorders; and broader, long-term interpersonal, social, educational, and occupational dysfunction [6]. Recent studies have shown that depression cases worldwide are increasing exponentially each year and are expected to become the leading cause of burdened illness worldwide by 2030 [7].

At present, the pathogenesis of depression remains unclear, and researchers have proposed several hypotheses, such as the reduction of monoamine neurotransmitter secretion [8] and the weakening of synaptic transmission efficiency [9]; the dysfunction of the hypothalamic–pituitary–adrenal cortex axis [10]; the interaction between stressful environments and genetic factors leading to neuroendocrine disorders; and the abnormal synaptic transmission, synaptic plasticity, and brain cortical and limbic system structural functional loop abnormalities [11]. With the deepening of research, more and more evidence has revealed that the neuroimmune system and inflammatory cytokines are involved in the production of depressive symptoms. The cell theory suggests that stress stimuli or overactive immune systems can produce inflammatory cytokines, which play an important role in the pathogenesis of depression [12,13]. As one of the regions with relatively rich neural circuit connections in the brain, the hippocampus is often associated with memory and information storage functions, and recent studies have also found that it can participate in the regulation of emotional and cognitive activities, especially in the pathogenesis of depression. Therefore, in recent years, many studies on the pathogenesis of depression have pointed to changes in hippocampal tissue [14]. As a result, anti-inflammatory therapy that reduces hippocampal inflammatory response and improves hippocampal function has become a new target for the prevention and treatment of depression.

Studies have shown that hippocampal neuronal injury is closely related to the nucleotide-binding oligomerization domain-like protein 3 (NLRP3) inflammasome [15]. NLRP3 is an important protein that mediates innate immunity in the human body and is a core component of the inflammasome [16]. NLRP3 can be activated by recognizing specific molecular patterns and binding to ligands, inducing the assembly of the NLRP3 inflammasome, and promoting the maturation of downstream interleukin (IL)-1β and IL-18 precursors, leading to impaired neuronal plasticity [17,18]. The NLRP3 inflammasome is regulated by reactive oxygen species (ROS), which are key activators that directly or indirectly trigger NLRP3 inflammasome activation by acting on NF-κB [19,20]. SIRT3 is a mainly mitochondrial NAD+-dependent deacetylase that maintains ROS homeostasis by targeting mitochondrial enzymes such as superoxide dismutase 2 (SOD2) [21], which converts harmful superoxide radicals into nontoxic oxygen or hydrogen peroxide [22,23].

Moderate aerobic exercise has been shown to improve the body’s anti-inflammatory capacity. Studies have demonstrated that aerobic exercise plays an important role in various neurodegenerative diseases, including Alzheimer’s disease, chronic cerebral ischemia, and stroke, by exerting significant anti-inflammatory effects [24,25]. Research has also suggested that both intermittent and continuous aerobic exercise can suppress the transduction of the IKKβ/NF-κB inflammatory pathway to a certain extent, regulate the secretion of inflammatory factors, and improve the body’s inflammatory response [26]. However, there are few experimental studies that have investigated whether exercise can intervene and regulate the pathological process of depression through the NLRP3 signaling pathway, particularly the SIRT3/ROS/NLRP3 axis [27,28]. This study aims to explore the effects of aerobic exercise on the expression of SIRT3 and its downstream pathways ROS/NLRP3 and the hippocampal inflammatory response in CUMS-induced depressed rats.

## 2. Materials and Methods

### 2.1. Experimental Materials

Healthy male SPF-level Sprague Dawley (SD) rats (animal production license number: SCXK (Chuan) 2020-0030) were used as experimental subjects, totaling 29 rats at 8 weeks old and weighing (224 ± 30) g, provided by Chengdu Dashuo Experimental Animal Co., Ltd. (Dashuo Co., Ltd. Chengdu, China). ELISA kits (Servicebio, Wuhan, China), RT-PCR reagent kits (Servicebio, Wuhan, China), NanoDrop2000 ultramicro spectrophotometer (Thermo, Waltham, MA, USA), fluorescent quantitative PCR instrument (BIORAD, critical, Hercules, CA, USA), and RT6100 automatic ELISA instrument (Rayto, Shenzhen, China) were used. The ethical approval process at our institution has been impacted by the COVID-19 pandemic, resulting in the unavailability of the original ethical approval number for this study. As an alternative arrangement, this research obtained approval from the Animal Center of Shaanxi Normal University. All participants in the experiments underwent relevant assessments organized by the Animal Center, and the protocols executed in this study were endorsed in the Animal Center’s admission agreement (Protocol Number: 20210823-1). The execution of the chronic unpredictable mild stress protocol, exercise protocol, and anesthesia protocol during the study was supervised by the Animal Center. All other animal care procedures were approved by the Animal Center of Shaanxi Normal University and conducted in accordance with the regulations and general recommendations outlined in the Chinese Laboratory Animal Management regulations.

### 2.2. Experimental Animal Treatment

#### 2.2.1. Experimental Animal Grouping

After adapting to the environment for one week, the rats were randomly divided into the following groups: blank control group with 9 rats (Control group, CON), chronic unpredictable mild stress (CUMS) model control group with 10 rats (CUMS group, CUMS), and exercise CUMS group with 10 rats (EX + CUMS). 

#### 2.2.2. Chronic Unpredictable Mild Stress (CUMS) Modeling Method

The chronic unpredictable mild stress method consisted of horizontal shaking, odor interference, tilted cage, tail clipping, reversed day and night cycle, food and water deprivation, wet bedding, binding, overcrowding, ice water swimming, and darkness [29]. In the day–night cycle reverse stimulus, the dark environment was realized with two layers of blackout cloth and the illumination condition was realized with large LED lights. Most of the stimulation takes place between 12 and 15 o’clock in the day. The 13 chronic stress stimuli were randomly applied to the rats, 1–2 stimuli per day, for a total modeling time of 5 weeks to induce depression-like symptoms while preventing adaptation to the stimuli (Table 1).

#### 2.2.3. Exercise Program

**Figure 1:**
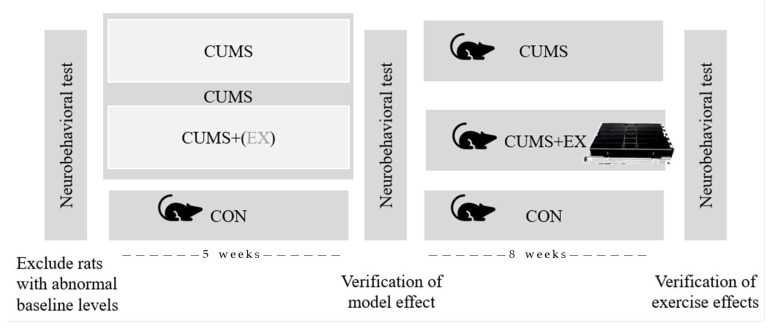
Experimental procedure diagram.

After 5 weeks of CUMS modeling, the EX + CUMS rats were subjected to an adaptive treadmill exercise program with a 0-degree slope, 15 m/min, 60 min/day, 6 days/week of moderate intensity for 8 weeks (Figure 1).

### 2.3. Neurobehavioral Assessment

Neurobehavioral testing for the final three groups was conducted after the exercise intervention was completed. However, neurobehavioral testing was also performed at the very beginning of the experiment to exclude rats with significantly abnormal baseline levels (e.g., most rats had sucrose preference indices higher than 95%, with abnormal rats typically in the 60–80% range). Neurobehavioral testing was also performed after the CUMS modeling to exclude the CUMS rats with high sucrose preference indices and high water struggle times. Since neither test was a comparison of three groups, the data are not presented. 

#### 2.3.1. Sucrose Preference Test

Used to determine the state of pleasure deficiency in animals. Prior to the experiment, rats were trained to adapt to 3% sugar-containing drinking water for 2 days. Before testing, all rats were deprived of water overnight (water deprivation time of over 12 h). Each rat was placed with a preweighed pure water bottle and a 2% sucrose water bottle. After 1 h, the relative positions of the two bottles were switched. The amount of sucrose and pure water consumed by the rats within 2 h was recorded, and the sucrose preference rate was calculated as sucrose consumption/(sucrose consumption + pure water consumption) × 100% for each individual rat.

#### 2.3.2. Forced Swimming Test

Rats were placed in a circular transparent water tank with a diameter of 25 cm and a water depth of 30 cm containing warm water (25 ± 1 °C). The rats were recorded for immobility using a high-definition camera for 6 min, and the immobility time during the last 4 min of the test was recorded [30]. In addition, because there were similar swimming stimuli in the CUMS modeling, the forced swimming experiment was not pretested.

### 2.4. Tissue and Sample Collection 

After the neurobehavioral assessments, for each group of 29 rats, pentobarbital sodium (dose: 50 mg/kg) was injected into the abdominal cavity for anesthesia, followed by cardiac blood collection and decapitation on ice to remove the scalp and fur. The skull was opened along the sagittal suture from the foramen magnum; the brain cortex was exposed by gently prying with glass forceps, and the hippocampus was exposed. The hippocampus was then separated from the surrounding brain tissue and used for ELISA detection, total RNA extraction, and ROS fluorescent staining. Three rats were randomly selected from each group and the hippocampus was removed using the same method described above, frozen at −80 °C, and used for western blot analysis of protein content.

### 2.5. ELISA

After thawing, the hippocampal tissue was homogenized on ice using an electric homogenizer, and the supernatant was collected after low-temperature centrifugation at 2500 r/min for 15 min. Standard samples were diluted and added strictly according to the ELISA kit instructions (Servicebio, Wuhan, China), and the processes of incubation, washing, enzyme addition, color development, and termination were carried out in sequence. The OD values of IL-1β and IL-18 in the hippocampal tissue of each rat were measured at 450 nm wavelength using an enzyme-linked immunosorbent assay (ELISA) reader after calibration, and the concentrations of each factor in the sample were calculated using the standard curve.

### 2.6. Real-Time Quantitative Reverse Transcription Polymerase Chain Reaction (RT-qPCR)

First, the electric homogenizer was used to homogenize the sample in Trizol, followed by centrifugation and 10 min of incubation at room temperature for sufficient lysis. Total RNA was extracted by strictly following the instructions of the kit and adding the reagents step by step, followed by centrifugation. The concentration and purity of the extracted total RNA were measured using a Q5000 ultraviolet spectrophotometer. Total RNA was reverse transcribed into cDNA and PCR amplification was performed using SYBR PCR mixture according to the product manual. The data was analyzed using the 2^−ΔΔCt^ method for relative quantification. The relevant primer design was conducted by Wuhan Saierwei Co., Ltd., Wuhan, China, as shown in the table.

### 2.7. Immuno-Fluorescent Staining for Reactive Oxygen Species (ROS)

The content of ROS in the hippocampus was detected using the fluorescent probe dihydroethidium (DHE). Hippocampal tissue stored at −80 °C was sliced into 5 μm sections using a cryostat. The DHE staining solution was added and incubated at room temperature for 15 min. Then, the slides were washed three times with PBS (pH = 7.4) while shaking for 5 min each time. After incubating with DAPI staining solution for 10 min at room temperature, the slides were washed three more times with PBS while shaking and then covered with coverslips. The slides were observed under a fluorescent microscope and photographed. The Image J software (version: 1.53k) was used for semiquantitative analysis of the red fluorescent-positive ROS staining area.

### 2.8. Western Blotting

Hippocampal tissue was taken out, minced, ground, and lysed. The hippocampal tissue was centrifuged at 12,000× *g* and 4 °C for 5 min, and the supernatant was collected. The protein concentration was determined using a microplate reader, and 100 μg of protein sample was loaded into a 5% SDS-polyacrylamide gel, followed by transfer onto a 0.45 μm pore size nitrocellulose membrane (NC membrane). The membrane was then blocked with 3% (*w*/*v*) skim milk in TBST buffer at 4 °C for 2 h. IL-1β and IL-18 were diluted 1:1000 with TBST buffer and incubated overnight. The membrane was washed five times with TBST for 5 min each time and incubated with antirabbit IgG secondary antibody conjugated with the detection reagent (diluted 1:2000–5000) at room temperature for 1 h. β-actin was used as an internal control. The gel imaging system was used for exposure and imaging of the film. The results were compared using the gray value ratios of SIRT3/β-actin and NLRP3/β-actin.

### 2.9. Statistical Analysis

Statistical analysis was performed using Graphpad8.0.2 software, and the data were expressed as mean ± standard deviation (M ± SD). After testing the homogeneity of variance for all data, one-way ANOVA variance analysis was performed, followed by post-hoc analysis using Tukey’s multiple comparison test. A significance level of *p* < 0.05 was considered as statistically significant.

## 3. Results

### 3.1. The Effects of Aerobic Exercise on Neurobehavioral Performance in CUMS-Induced Depressed Rats 

Neurobehavioral assessment results showed that compared to the CON group the sucrose preference index significantly decreased and forced swimming time significantly increased in the CUMS group and EX + CUMS group rats (*p* < 0.05). After 8 weeks of aerobic exercise training, the sucrose preference index significantly increased and struggling time in water significantly decreased in the EX + CUMS group rats (*p* < 0.05) (Table 2).

### 3.2. The Effects of Aerobic Exercise on ROS Levels in CUMS-Induced Depressed Rats

**Figure 2:**
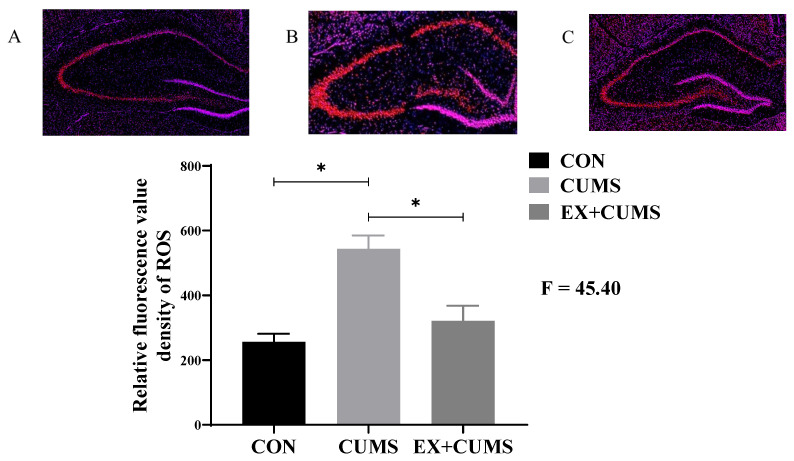
The effects of aerobic exercise on ROS levels in CUMS-induced depressed rats. Note: (**A**) indicates CON group, (**B**) indicates CUMS group, and (**C**) indicates EX + CUMS group. * Indicates that the difference between the two groups is significant.

Immunofluorescence staining results showed that compared to the CON group, ROS levels in the hippocampus of the CUMS group rats significantly increased (*p* < 0.05). After aerobic exercise intervention, ROS levels in the hippocampus tissue of the EX + CUMS group rats were significantly suppressed, and the difference between the EX + CUMS group and CUMS group was statistically significant (*p* < 0.05) (Figure 2).

### 3.3. The Effects of Aerobic Exercise on mRNA Expression in CUMS-Induced Depressed Rats

**Figure 3:**
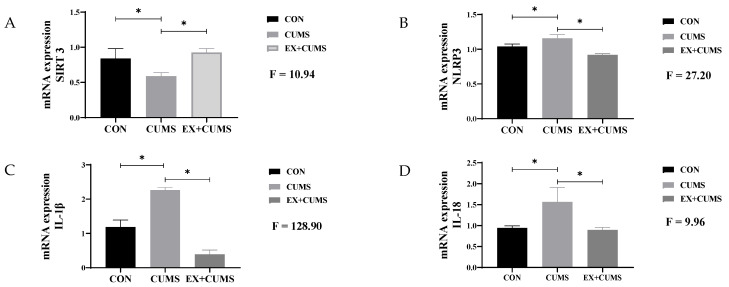
The effects of aerobic exercise on mRNA expression in CUMS-induced depressed rats. Note: (**A**) indicates SIRT3, (**B**) indicates NLRP3, (**C**) indicates IL-1β, (**D**) indicates IL-18. * Indicates that the difference between the two groups is significant.

Compared to the CON group, chronic stress stimulation increased the mRNA expression of NLRP3, IL-1β, and IL-18 and decreased the mRNA expression of SIRT3 in the hippocampus tissue of rats. Aerobic exercise was able to inhibit the mRNA expression of NLRP3, IL-1β, and IL-18 and enhance the mRNA expression of SIRT3 (Figure 3).

### 3.4. The Effects of Aerobic Exercise on Protein Expression in the Hippocampus Tissue of CUMS-Induced Depressed Rats 

**Figure 4:**
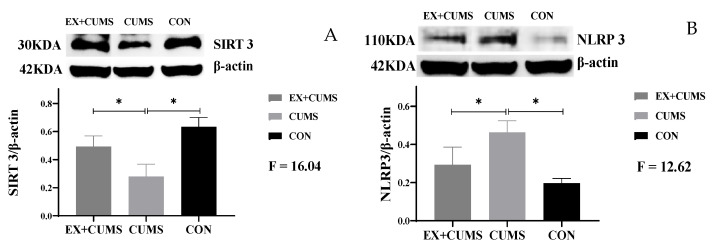
The effects of aerobic exercise on protein expression in the hippocampus tissue of CUMS-induced depressed rats. Note: (**A**) indicates SIRT3, (**B**) indicates NLRP3. * Indicates that the difference between the two groups is significant.

Compared to the CON group, chronic stress stimulation increased the protein content of NLRP3 and decreased the protein content of SIRT3 in the hippocampus tissue of rats (*p* < 0.05). Aerobic exercise attenuates NLRP3 protein overexpression in depressed rats (*p* < 0.05) (Figure 4).

### 3.5. The Effects of Aerobic Exercise on Inflammatory Cytokine Levels in the Hippocampus Tissue of CUMS-Induced Depressed Rats 

**Figure 5:**
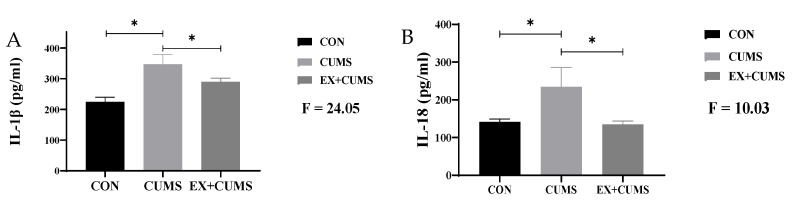
The effects of aerobic exercise on inflammatory cytokine levels in the hippocampus tissue of CUMS-induced depressed rats. Note: (**A**) indicates IL-1β, (**B**) indicates IL-18. * Indicates that the difference between the two groups is significant.

Compared to the CON group, the levels of inflammatory cytokines in the hippocampus tissue of CUMS group rats significantly increased (*p* < 0.05). After aerobic exercise intervention, the levels of IL-1β and IL-18 inflammatory cytokines in the hippocampus tissue of rats was significantly suppressed, and the difference between the EX + CUMS group and CUMS group was statistically significant (*p* < 0.05) (Figure 5).

## 4. Discussion

This study found that exercise can promote SIRT3 expression and exert antioxidant and anti-inflammatory effects to improve depression-like behavior. Specifically, exercise can increase SIRT3 levels in the hippocampal tissue, reduce the production of ROS, and thus lower the level of cellular oxidative stress to exert antioxidant effects. In addition, SIRT3 can also regulate the inflammatory response and reduce the expression of inflammatory factors to exert anti-inflammatory effects. Therefore, the results of this study further support the view that exercise improves depression by upregulating SIRT3 expression to exert anti-inflammatory effects.

As a member of the mitochondrial protein deacetylase family, SIRT3 has been extensively studied for its roles in cell metabolism and inflammation. The results of this study indicate that aerobic exercise upregulates the expression of SIRT3, which is consistent with previous research findings. Many studies have shown that aerobic exercise can increase the expression level of SIRT3. Several studies found that aerobic exercise significantly increases the expression of SIRT3 in mouse heart [31] and skeletal muscle [32], and this increase is related to the improvement of mitochondrial function. Another study found that long-term high-intensity interval training can enhance SIRT3 protein expression in human skeletal muscle [33]. This study confirms that exercise can upregulate SIRT3 expression in the hippocampal tissue of depressed rats. The impact of exercise on SIRT3 expression may be influenced by the form and duration of exercise, as well as the subject’s state. Studies have shown that different types of exercise have varying effects on the expression level of SIRT3, with running and swimming having a more significant effect on SIRT3 expression than static load training [34,35]. It is worth noting that although most studies support the idea that exercise can increase SIRT3 expression, there are also some studies that have reached the opposite conclusion. For example, two studies found that exercise does not increase SIRT3 expression in muscle tissue in fasting people or for those who only engage in short-term exercise [36,37]. Overall, most studies support the idea that exercise can increase SIRT3 expression, and the mechanism behind this may be related to the mitochondrial biogenesis and improvement of mitochondrial function induced by exercise.

The results of this study also indicate that SIRT3 has a regulatory effect on ROS and NLRP3 inflammasomes. It was found that upregulation of SIRT3 can inhibit ROS production and regulate the activation and inflammatory response of NLRP3 inflammasomes, thereby exerting anti-inflammatory effects. This is consistent with previous research findings [38]. Other studies have also confirmed the regulatory effects of SIRT3 on ROS and NLRP3. Song et al. showed that downregulation of SIRT3 leads to an increase in ROS production, triggering mitochondrial oxidative stress and cell apoptosis, as well as an increase in the activation of NLRP3 and expression of inflammatory cytokines [39]. In contrast, Xia et al. demonstrated that upregulation of SIRT3 can suppress the activation of NLRP3 inflammasomes and the expression of inflammatory cytokines by regulating mitochondrial membrane potential to reduce ROS production [28]. These research results are consistent with the findings of this study, indicating that SIRT3 can regulate the activation and inflammatory response of ROS and NLRP3 inflammasomes through various mechanisms. However, SIRT3 may interact with other signaling pathways to regulate cellular inflammation. In the future, further studies are needed to elucidate the mechanism of action of SIRT3 and its role in diseases such as depression, in order to better understand its biological functions and provide a basis for the development of relevant therapeutic strategies.

The pathogenesis of depression is not fully understood. In recent years, increasing evidence suggests that inflammatory responses play an important role in the occurrence and development of depression [40]. The results of this study indicate that inflammation factors, such as ROS and NLRP3, play an important role in depression. ROS is a type of free radical that can bind with molecules such as cell membranes, DNA, and proteins causing oxidative damage and resulting in inflammatory reactions [41]. NLRP3 is a protein in the inflammasome that induces inflammatory responses and is involved in the occurrence and development of various diseases [16]. This study shows that inflammation factors such as ROS and NLRP3 are significantly upregulated in the hippocampal tissue of depressive mice, indicating that they play an important role in the occurrence and development of depression. Studies have shown that the lack of anti-inflammatory factors is closely related to the occurrence and development of depression [42,43]. Therefore, inhibiting inflammatory responses and promoting anti-inflammatory responses are important strategies for treating depression. Exercise, as a non-pharmacological treatment method, has been widely studied for its anti-inflammatory effects [44]. Exercise can inhibit inflammatory responses and promote anti-inflammatory responses through various pathways, and multiple signaling pathways are involved in regulating anti-inflammatory effects, such as NF-κB and JAK/STAT [45,46]. NF-κB is a transcription factor that is widely regarded as the main regulator of inflammatory responses. The JAK-STAT pathway is an important signaling pathway, and some antidepressants can also exert their effects by regulating the JAK-STAT pathway [47]. Other important proteins involved in the signaling pathways of hippocampal inflammation include MAPK and SIRT1 [48,49]. This study confirms that SIRT3 in the SIRT family also has anti-depressive effects, mainly by inhibiting the activation of ROS and NLRP3 inflammasomes and exerting anti-inflammatory effects. The survival and death of neurons, and the occurrence and progression of neuroinflammatory responses are regulated by multiple signaling pathways, and many unresolved questions remain regarding the roles of these proteins and signaling pathways, requiring further research.

This study also has certain limitations. Firstly, it was conducted only in animal models, and more research is needed to confirm the reproducibility and applicability of these findings in humans. This study mainly focused on the SIRT3/ROS/NLRP3 signaling pathway in the hippocampus, but other signaling pathways may also be involved in the formation of depression-like behavior, such as the NF-κB signaling pathway which is closely related to NLRP3 and requires further investigation [50]. In addition, differences in exercise type and intensity may affect its impact on SIRT3 in the hippocampus, therefore more research is needed to determine the most effective exercise method.

## 5. Conclusions

This study found that exercise can increase SIRT3 in the hippocampus, reduce ROS production, regulate inflammatory responses, and lower the expression of inflammatory factors, thus exerting an antidepressant effect. Therefore, the results of this study further support the view that exercise ameliorates depressive-like symptoms and regulates some proteins up or downregulated by CUMS. In the future, further studies are needed to investigate the mechanism of SIRT3 and its role in diseases such as depression, in order to better understand its biological function and provide a basis for developing related therapeutic strategies.

## Figures and Tables

**Table 1:** Chronic Unpredictable Mild Stress Modeling Method.

	Week 1	Week 2	Week 3	Week 4	Week 5
Day 1	Horizontal shaking odor interference	Reversed day and night cycle	Horizontal shaking odor interference	Reversed day and night cycle	Horizontal shaking odor interference
Day 2	Tilted cage Tail clipping	Wet bedding	Tilted cage Tail clipping	Wet bedding Single cage	Tilted cage Tail clipping
Day 3	Reversed day and night cycle	Horizontal shaking odor interference	Wet bedding	Horizontal shaking odor interference	Reversed day and night cycle
Day 4	water deprivation	Food deprivation	water deprivation	Food deprivation	water deprivation
Day 5	Overcrowding Ice water swimming	Tilted cage Tail clipping	Darkness	Tilted cage Tail clipping	Overcrowding Ice water swimming
Day 6	Wet bedding Noise stimulation	Ice water swimming	Binding Ice water swimming	Ice water swimming	Wet bedding Binding
Day 7	Food and water deprivation	Food and water deprivation	Food and water deprivation	Food and water deprivation	Food and water deprivation

**Table 2:** The effects of aerobic exercise on neurobehavioral performance in CUMS-induced depressed rats.

Groups	n	SP (%)	FSI (s)
CON	9	88.94 ± 1.79	49.45 ± 25.46
CUMS	10	72.41 ± 2.20 *	139.60 ± 31.62 *
EX + CUMS	10	87.09 ± 4.58 #	73.20 ± 45.08 #
F		175.923	32.106
P		0.000	0.000

Note: SP refers to sucrose preference; FSI refers to forced swimming immobility; * Compared with CON group, *p* < 0.05; # Compared with CUMS group, *p* < 0.05.

## Data Availability

Data are available by contacting the corresponding author upon reasonable request.
